# Dejerine-Sottas disease: a case report

**DOI:** 10.1590/S1516-31802003000500006

**Published:** 2003-09-01

**Authors:** Jaqueline Luvisotto Marinho, José Luis Alonso Nieto, Edenilson Eduardo Calore

**Keywords:** Hereditary peripheral neuropathies, Dejerine-Sottas disease, Nerve biopsy, Genetic neuropathies, Neuropatias periféricas hereditárias, Doença de Dejerine-Sottas, Biópsia de nervo, Neuropatias genéticas

## Abstract

**CONTEXT:**

Hereditary peripheral neuropathies (hereditary motor-sensory neuropathies or hereditary demyelinating neuropathies) are abnormalities of Schwann cells and their myelin sheaths, with peripheral nerve dysfunction. They include Charcot-Marie-Tooth disease, Dejerine-Sottas disease, congenital hypomyelinating neuropathy and hereditary neuropathy with liability to pressure palsy.

**OBJECTIVE:**

The objective of the present work was to describe a case of Dejerine-Sottas disease.

**CASE REPORT:**

A 9-year-old boy presented progressive slight motor deficit in the lower limbs, particularly in the feet, and generalized hyporeflexia. Electromyography disclosed significant reduction in motor and sensory nerve conduction velocities. Sural nerve biopsy showed axons surrounded by a thin myelin sheath and concentrically arranged cytoplasmic processes of Schwann cells forming onion-bulbs. No axon damage was observed.

## RESUMO

**CONTEXTO:**

As neuropatias periféricas hereditárias ou neuropatias hereditárias desmielinizantes são anormalidades das células de Schwann e de suas bainhas de mielina com disfunção dos nervos periféricos. Estas neuropatias incluem: doença de Charcot-Marie-Tooth, doença de Dejerine-Sottas, neuropatia hipomielinizante congênita e neuropatia hereditária com predisposição a paralisias por pressão.

**OBJETIVO:**

Descrever um caso de doença de Dejerine-Sottas.

**RELATO DE CASO:**

Menino com nove anos de idade apresentou perda motora leve progressiva em membros inferiores, principalmente nos pés, e hiporreflexia generalizada. A eletromiografia revelou redução importante das velocidades de condução nervosa motora e sensitiva. A biópsia de nervo sural mostrou axônios circundados por uma fina bainha de mielina e pelos processos citoplasmáticos das células de Schwann dispostos concentricamente, formando bulbos de cebola (*onion bulbs*). Não foi observado dano axonal.

## INTRODUCTION

Hereditary motor-sensory neuropathies are abnormalities of axons or Schwann cells and their myelin sheaths, with peripheral nerve dysfunction. The diagnosis of these neuropathies is based on clinical and ancillary examination. Electromyography generally reveals decreased nerve conduction velocity. Sural nerve biopsy reveals demyelination and remyelination features, with indications of Schwann cells disturbances. Genetic studies are of value not only for diagnosing these diseases, but also for better understanding the molecular events that result in the clinical symptoms.

The classification of hereditary motor-sensory neuropathies depends on the clinical and laboratory features. Charcot-Marie-Tooth disease can be divided into two types: Charcot-Marie-Tooth type 1/hereditary motor-sensory neuropathy type I (demyelinating) and Charcot-Marie-Tooth type 2/hereditary motor-sensory neuropathy type II (axonal), based on electrophysiological studies. Hereditary motor-sensory neuropathy type III is also called Dejerine-Sottas disease. Congenital hypomyelinating neuropathy is considered to be a rare and severe form of Dejerine-Sottas disease, and is thought to reflect dysmyelination rather than demyelination. Other types include hereditary neuropathy with liability to pressure palsy and other rare forms of demyelinating peripheral neuropathies.

Four genes have been identified that are related to these disorders: peripheral myelin protein 22, myelin protein zero, gap junction protein connexin 32 and early growth responsive gene 2.

The purpose of the present work was to describe a case of Dejerine-Sottas disease.

## CASE REPORT AND NERVE BIOPSY

A 6-year-old boy was presenting progressive reduction of strength in the lower limbs associated with posture difficulties. Neurological examination at the age of 9 years revealed slight motor deficit in the lower limbs, particularly in the feet, and generalized hyporeflexia. There were thickenings of the ulnar and sural nerves. Electromyography showed significant reduction in motor and sensory nerve conduction velocities: ulnar, median, radial, tibial and sural. Electromyography performed on the boy's parents gave normal results. These clinical and electrophysiological features are suggestive of hereditary motor-sensory neuropathy.

Sural nerve biopsy was performed at the level of the left lateral malleolus. A fragment of length 3 cm was fixed in glutaraldehyde 2% in phosphate buffer and post-fixed in osmium tetroxide and uranyl acetate, and then embedded in resin for electron microscopy. Semi-thin sections and ultra-thin sections were cut.

**Figure 1:**
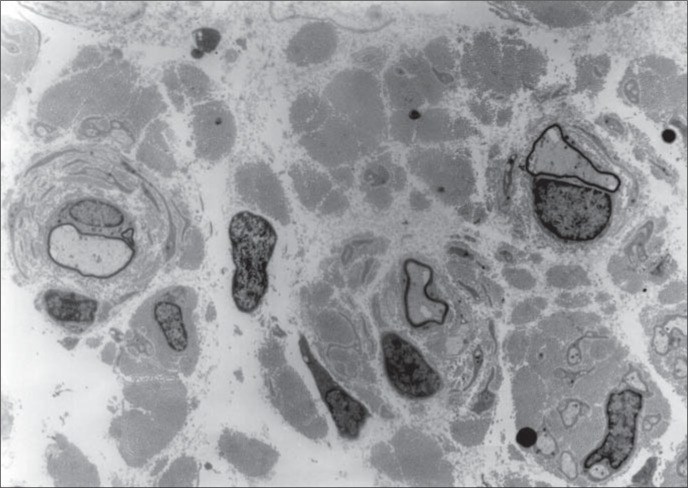
Three axons surrounded by a thin myelin sheath and concentrically arranged cytoplasmic processes of Schwann cells forming onion-bulbs. Note the thickened bands of collagen interposed among these axons (2,500 x).

**Figure 2:**
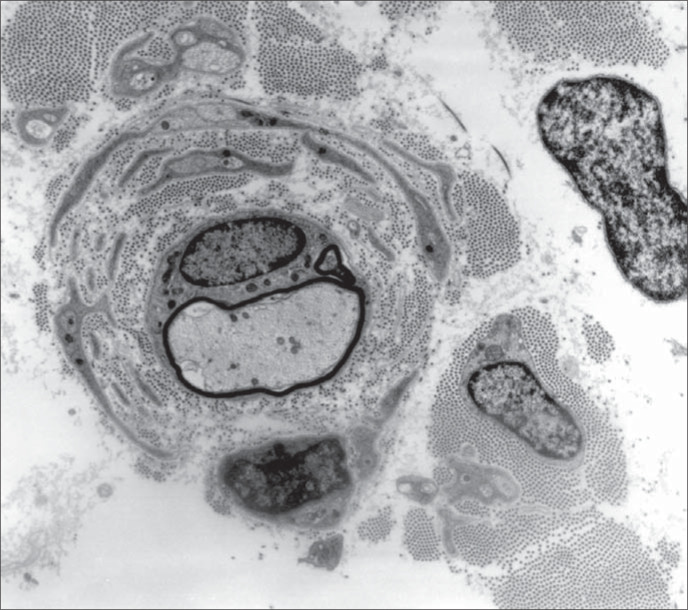
Higher magnification of the preceding figure showing one axon surrounded by a thin myelin sheath and concentrically arranged cytoplasmic extensions of Schwann cells forming onion-bulbs. Observe the bands of collagen interposed among these Schwann cells extensions (5,000 x).

**Figure 3:**
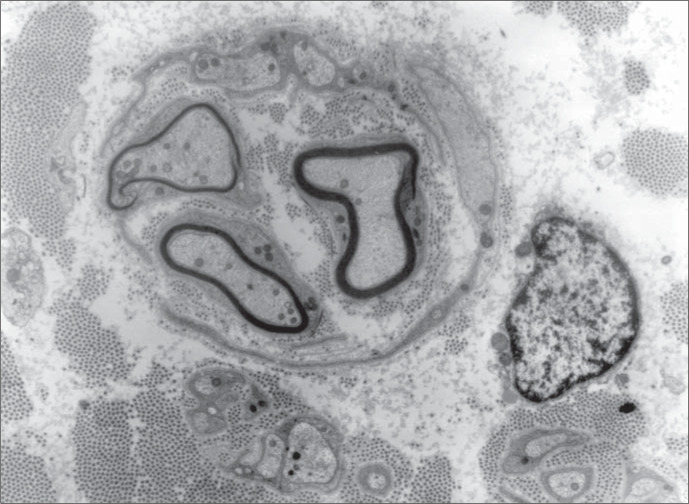
Three axons surrounded by a group of Schwann cells and their concentrically arranged processes. Each axon is individually surrounded by a myelin sheath (5,000 x).

It was observed that the sural ner ve was thickened. There was increased conjunctive tissue with thickened bands of collagen fibers in the endoneurium. All the axons observed were surrounded by a thin myelin sheath and concentrically arranged cytoplasmic processes of Schwann cells forming onion-bulbs. These onion-bulbs were observed in practically all axons. Collagen bands were often interposed among these Schwann cells extensions. Sometimes, the same group of Schwann cells and their concentrically arranged processes surrounded two or three axons individually wrapped in myelin sheaths. No axon damage was observed (Figures 1, 2 and 3).

## DISCUSSION

The clinical symptoms of Charcot-Marie-Tooth patients normally appear in the first or second decade of life. However, the electrophysiological characteristics can be identified before the onset of clinical symptoms, and usually by the age of 2 years. Muscle weakness starts in the feet and legs. Later on, the foot drops with each step, forcing the patient to lift the knee, thus resulting in step-page or equine gait. Atrophy of the legs due to wasting of the peroneal muscles can result in a stork leg or inverted champagne bottle appearance. Claw foot deformity develops with age. Weakness of the intrinsic hand muscles generally occurs late in the course of the disease but is not usually related to the degree of leg weakness or atrophy or to the age of the patient. Muscle stretch reflexes disappear early in the ankles and later in the patella and upper limbs. In general, sensory symptoms are restricted to decrease in vibra-tory sense.^1^

Glocker et al. (1999)^2^ showed that involvement of the facial nerve is common in Charcot-Marie-Tooth type 1 and DejerineSottas disease, but this was not associated with clinical dysfunction of facial muscles. Char-cot-Marie-Tooth type 1 exhibits moderately to severely reduced motor nerve conduction velocities (< 40 m/s). The conduction deficit in Charcot-Marie-Tooth type 1 is bilaterally symmetrical, which suggests intrinsic Schwann cell defect.

Dejerine-Sottas disease shares considerable clinical, electrophysiological and pathological characteristics with Charcot-Marie-Tooth type 1. Onset in infancy, as seen by delays in motor milestones, severity of its features and motor nerve conduction velocities of < 10 m/s are hallmarks of this disorder.^1^

Dejerine-Sottas disease was initially thought to be inherited as an autosomal recessive trait. However, several mutations of dominant inheritance in the peripheral myelin protein 22 gene and the peripheral myelin protein zero gene have been reported in patients with Dejerine-Sottas disease. There are cases of autosomal recessive inheritance as originally defined by Dejerine and Sottas in 1893^3^ (Stogbauer et al., 1998^4^).

The nerve pathology of Dejerine-Sottas disease patients is similar to that found in Charcot-Marie-Tooth type 1 patients, but like the clinical symptoms, the findings are more severe, with thinner myelin sheaths and more unmyelinated fibers. Onion bulb formations are always seen in Dejerine-Sottas disease patients, often with double basal laminae.^1^

## References

[1] Warner LE, Garcia CA, Lupski JR (1999). Hereditary peripheral neuropathies: clinical forms, genetics, and molecular mechanisms. Annu Rev Med.

[2] Glocker FX, Rosler KM, Linden D, Heinen F, Hess CW, Lucking CH (1999). Facial nerve dysfunction in hereditary motor and sensory neuropathy type I and III. Muscle Nerve.

[3] Dejerine J, Sottas J (1893). Sur la névrite interstitielle, hypertrophique et progressive de l'enfance. Mem Soc Biol.

[4] Stogbauer F, Young P, Wiebusch H (1998). Absence of mutations in peripheral myelin protein-22, myelin protein zero, and connexin 32 in autosomal recessive Dejerine-Sottas syndrome. Neurosci Lett.

